# Improved and sustained triage skills in firemen after a short training intervention

**DOI:** 10.1186/s13049-015-0162-7

**Published:** 2015-10-20

**Authors:** Abraham Nilsson, Kristian Åslund, Maria Lampi, Helene Nilsson, Carl-Oscar Jonson

**Affiliations:** Centre for Teaching & Research in Disaster Medicine and Traumatology, Department of Clinical and Experimental Medicine, Linköping University, SE-581 85 Linköping, Sweden

**Keywords:** Triage, Triage training, SALT Mass Casualty Triage Algorithm, Trauma cards, Direct instruction, Firemen

## Abstract

**Background:**

A previous study has shown no measurable improvement in triage accuracy among physicians attending the Advanced Trauma Life Support (ATLS) course and suggests a curriculum revision regarding triage. Other studies have indicated that cooperative learning helps students acquire knowledge.

**Objective:**

The present study was designed to evaluate the effectiveness of trauma cards in triage training for firemen.

**Methods:**

Eighty-six firemen were randomly assigned into two groups: the trauma card group and the direct instruction group. Both groups received the same 30-min PowerPoint lecture on how to perform triage according to the Sort Assess Lifesaving interventions Treatment and transport (SALT) Mass Casualty Triage Algorithm. In the trauma card group, the participants were divided into groups of 3–5 and instructed to triage 10 trauma victims according to the descriptions on the trauma cards. In the direct instruction group, written forms about the same 10 victims were used and discussed as a continuation of the PowerPoint lecture. Total training time was 60 min for both groups. A test was distributed before and after the educational intervention to measure the individual triage skills. The same test was applied again 6 months later.

**Results:**

There was a significant improvement in triage skills directly after the intervention and this was sustained 6 months later. No significant difference in triage skills was seen between the trauma card group and the direct instruction group. Previous experience of multi-casualty incidents, years in service, level of education or age did not have any measurable effects on triage accuracy.

**Conclusions:**

One hour of triage training with the SALT Mass Casualty Triage Algorithm was enough to significantly improve triage accuracy in both groups of firemen with sustained skills 6 months later. Further studies on the first assessment on scene versus patient outcome are necessary to provide evidence that this training can improve casualty outcome. The efficacy and validity of trauma cards for disaster management training need to be tested in future studies.

## Introduction

Mass casualty incidents (MCIs) occur each year in our society [1]. Regardless of the cause, severely injured casualties instantly create a major need for medical assessment and care. Disasters and MCIs are often characterized by discrepancy between immediate needs and available resources. This discrepancy may be due to quantitative and/or qualitative scarcity in human or physical resources. In these situations, medical and organizational prioritization is essential in order to minimize mortality, morbidity and other indirect effects, and the prehospital care provider must shift their focus from an individual perspective to instead provide the greatest good for the greatest number [2, 3]. This is achieved through the process of triage, which is perhaps the single most important medical intervention to minimize morbidity and mortality [4].

Firemen usually arrive at the scene of an incident in large units and are able to access victims before paramedics and other medical staff. The rescue services arrive before the ambulance service in 64 % of incidents according to incidents reports from 2004 to 2013 provided by the Swedish Contingencies Agency (MSB). To support effective coordination, communication and task sharing between personnel, it is essential that they share a common basis with regard to triage because the accuracy of triage decisions may be an important factor in the effectiveness of the whole medical response [3]. Inaccuracy may lead to under triage, defined as the faulty assignment of an immediate care survivor to a delayed or minimal care category, or to over triage, defined as the faulty assignment of a delayed or minimal care survivor to immediate care [5].

Pedagogic studies have indicated that working in groups in an interactive manner helps students to acquire knowledge [6, 7]. Recently, an interactive teamwork concept has been developed for triage training in concordance with the Emergo Train System (ETS). This concept utilizes trauma cards, which are intended to be used as a pedagogic tool in triage training for prehospital care providers. Each trauma card represents one patient and the information that can be obtained at first glance is on the front of the card and information about what is found after a primary assessment is on the back.

Moreover, by measuring physicians’ triage performance before and after the Advance Trauma Life Support (ATLS) course, a previous study reported that the course using the ABCDE structure had no measurable effect on triage accuracy. Actions to increase triage skill are warranted [8].

Firemen were chosen for this study because they are often dispatched as one of the first units according to the standard response from emergency call centers. In many countries, firemen are well trained in basic prehospital and trauma life support focusing on teamwork for extrication and lifesaving assessment for one trauma patient [9]. However, as firemen frequently arrive first at the scene and, while awaiting ambulance personnel, they might need to do the first triage and assessment; under those circumstances, the firemen will make the first early and time-critical decisions about triage. Several triage algorithms exist but evidence to support one algorithm over another is limited [10–12]. To address this problem, the Centers for Disease Control and Prevention (CDC) in the United States proposed a new triage guideline entitled SALT (Sort, Assess, Lifesaving interventions, Treatment and/or transport) [11]. After the terrorist attack in Norway in 2011, the Norwegian Directorate of Health proposed a new guideline for use in MCIs based on SALT [13]. The SALT algorithm was therefore chosen for this study.

As group work seems to facilitate learning, triage training in groups using trauma cards would hypothetically result in better triage accuracy compared to triage trained with direct instruction. The aim of this study was to investigate triage skills and retention 6 months after an intervention comparing trauma card-based training (ETS) versus direct instruction.

## Methods

The present study was designed as a prospective randomized controlled trial of triage test scores before and after a triage lecture for 30 min and triage exercises for 30 min for two study groups. One group received trauma card-based training and the other group received triage training based on direct instruction. Henceforth, they are referred to the trauma card group and the direct instruction group, respectively.

### Participants

Sixteen firemen shifts located at four rescue stations in two Swedish municipalities were asked through their superiors to participate voluntarily in the study. Eight trauma card sessions and eight direct instruction sessions were set up to reach all 16 shifts. All shifts were randomized to either trauma card-based training or direct instruction using the randomization function in Microsoft Excel 2010 (Microsoft, Redmond, WA, USA v14.0.7153.5000). Before starting, all participants were informed in both verbal and written form about the purpose of the study and their ability to withdraw at any time without explanation. All participants verbally consented to participate in the study on those conditions. Demographic data such as age, educational background, years in service and multi-casualty experience were collected. All sessions took place during October, 2013.

### Triage lecture

A Microsoft PowerPoint 2010 (Microsoft, Redmond, WA, USA v14.0.7153.5000) lecture (18 slides, 30 min) on SALT triage based on the disaster medicine literature and published research papers [11, 14] was created in collaboration with national trauma instructors and disaster medicine experts. The aim, goals and learning objectives were defined including the history and purpose of triage, the origin of SALT triage and the SALT triage algorithm. Ten trauma victims with different injuries were chosen from the ETS patient bank. According to the SALT algorithm, the victims consisted of three red priority 1 victims with an Injury Severity Score (ISS) >15, three yellow priority 2 victims (ISS 8–12), three green priority 3 victims (ISS <3) and one black victim (dead on scene).

**Fig. 1:**
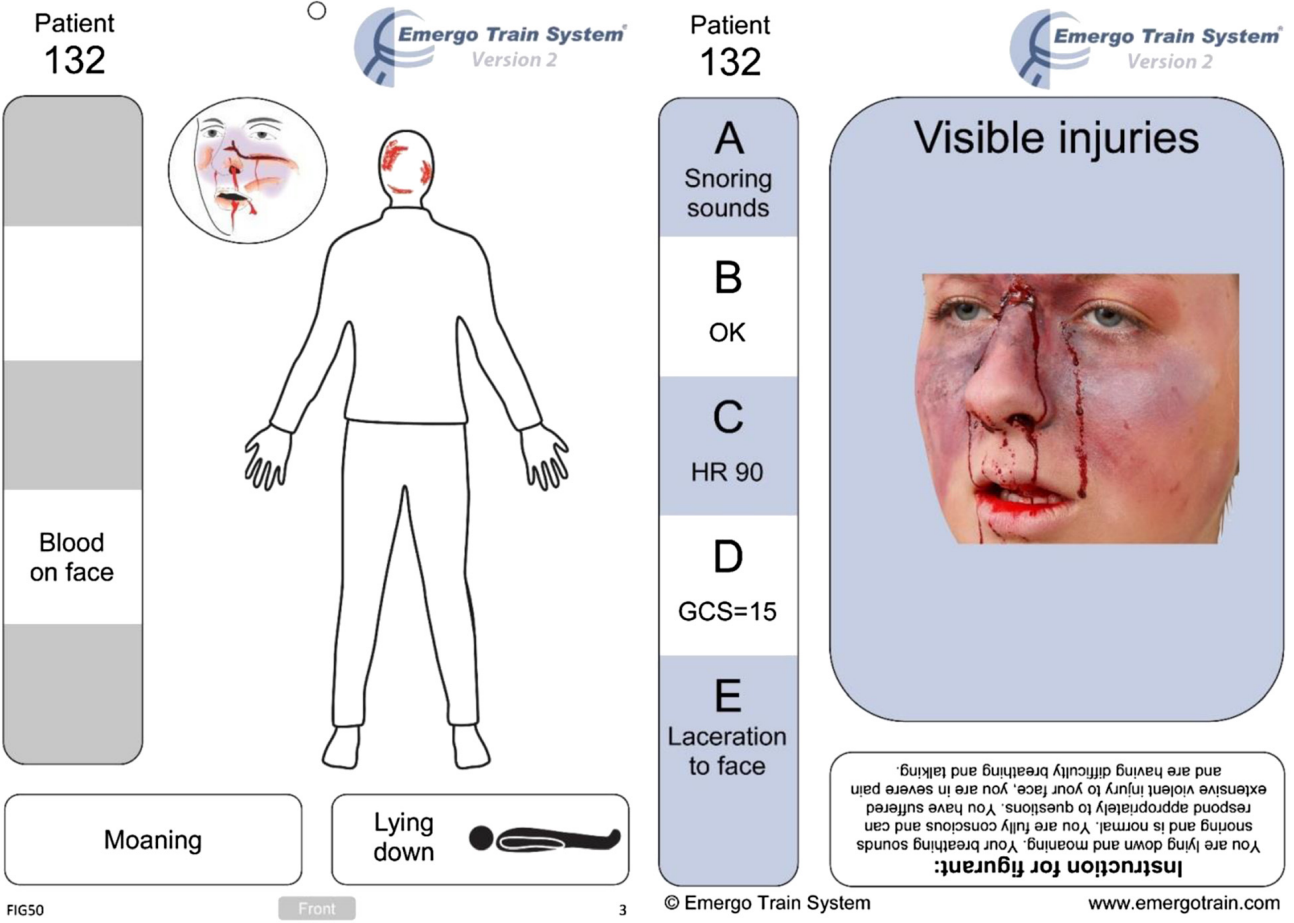
ETS Trauma card

Both study groups attended the 30-min PowerPoint lecture. In the trauma card sessions, the participants were told in groups of 3–5 people, to triage 10 patients. Information was given to them in form of trauma cards (Fig. 1). First, all patients were triaged according to their visible injuries. When consensus was reached in the groups, the patients were triaged again according to their vital signs and the additional findings after the primary survey. When all groups were satisfied with their triage decisions, the presenter discussed the correct triage category for each patient.

**Fig. 2:**
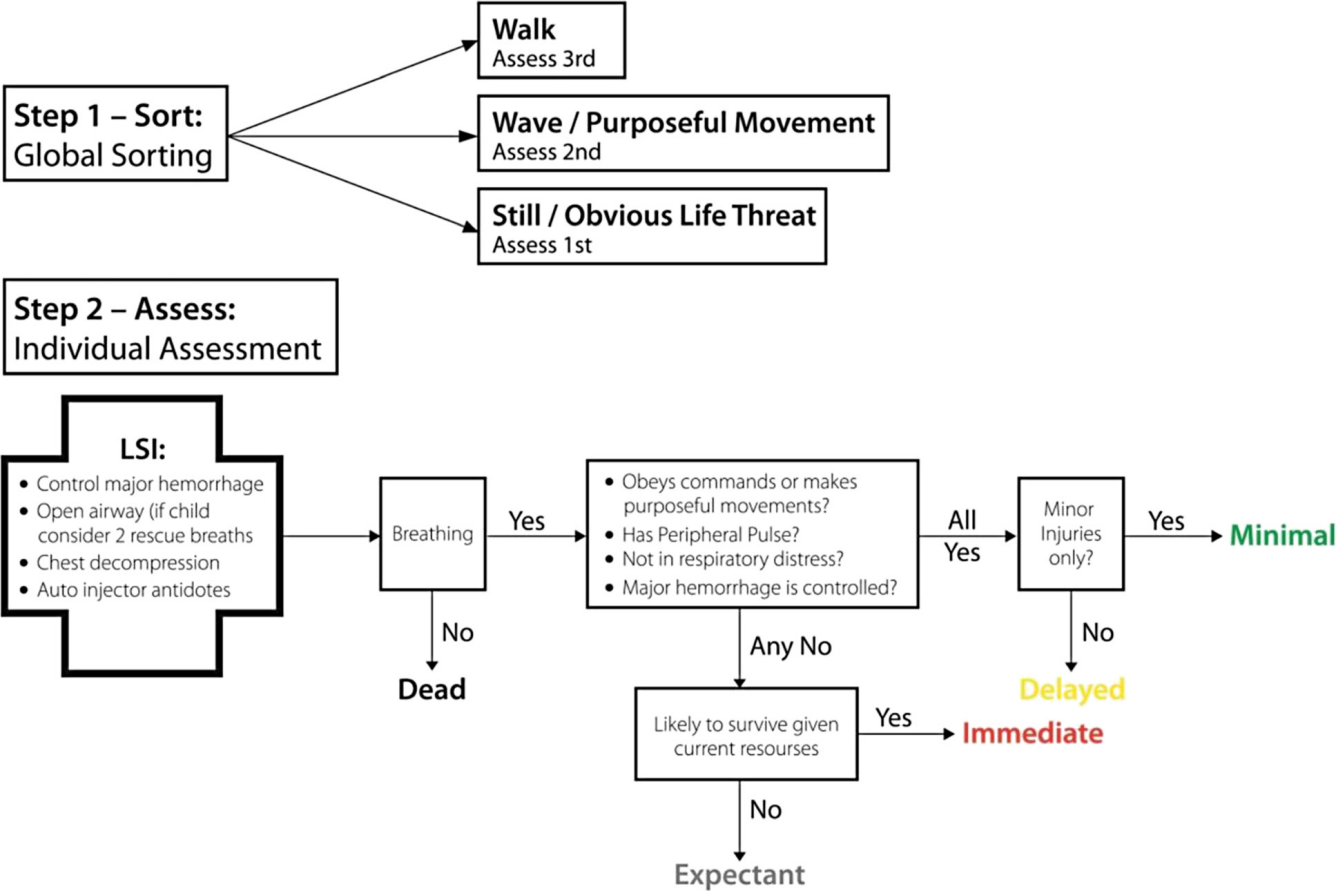
The SALT triage guideline. LSI, lifesaving intervention; SALT, Sort Assess Lifesaving intervention Treatment/transport [9]

In the direct instruction sessions, the same 10 patients were individually presented without the trauma cards by the presenter as a continuation of the lecture. In both groups, the starting condition was a bus crash with three ambulances on site and more on the way. To facilitate learning, a poster of the SALT triage algorithm was available for both groups during the patient scenarios (Fig. 2). The time for discussing patients was restricted to 30 min in both groups making the whole session 60 min. All lectures were held by the same presenter with a passive observer to ensure conformity. To further minimize bias, all participants were asked not to talk about the study with colleagues before all teaching sessions were completed.

### Data collection

To maximize the number of participants, and according to an agreement with the fire departments, a test encompassing 15, for the participants new, casualties used in earlier studies was used to measure triage performance [7, 15]. The same test was distributed to all participants before and after the training sessions. By not informing the participants of this arrangement, the incentive to memorize the pretest was minimized. The score was calculated as the number of correctly assigned triage categories. A time limit of 15 min to complete the test was set.

Six months after the initial training, a follow-up study was performed. All firemen who had participated in the first session were asked to take part. The follow-up did not involve any training. The same test with the 15 casualties was used, again under a time restriction of 15 min.

Exclusion criterion was a call out exceeding 45 min during any test or lecture. No participant was excluded due to this criterion. The data were stored on paper and unprocessed until data collection was complete. Microsoft Excel 2010 was then used to store the data in digital form. All participants and study groups were coded to ensure anonymity and were blinded to the analyser.

### Ethics, consent and permissions

The Regional Ethical Review Board in Linköping was consulted regarding ethical approval. The study was not required to undergo a formal approval process. Informed consent was obtained from all study participants. No remuneration was given to any participant.

### Statistical analysis

Calculations were blinded and made using IBM SPSS Statistics for Windows (Ver. 21.0 IBM Corporation, Armonk, NY). The Shapiro-Wilks test of normality was used. Because the tests before and after the training did not show normality, the Wilcoxon signed rank test was used to compare the scores at baseline and after the intervention as well as the post-test scores and the follow-up scores; the Mann–Whitney *U* test was used to investigate if previous experience of multi-casualty incidents, years in service, level of education or age or educational model was related to improvement or retention. A *P* value less than 0.05 was considered statistically significant. Due to the exploratory nature of the subgroup analyses, no correction for multiplicity was done. All data are presented as the median [interquartile range (IQR)].

## Results

Eighty-six firemen completed the tests before and after the training. Fifty-one participants completed the test at the 6-month follow-up. All participants were included in the analysis and missing data were treated as other triage categorization errors, although this did not contribute to over or under triage.

### Pretest and post-test scores

**Fig. 3:**
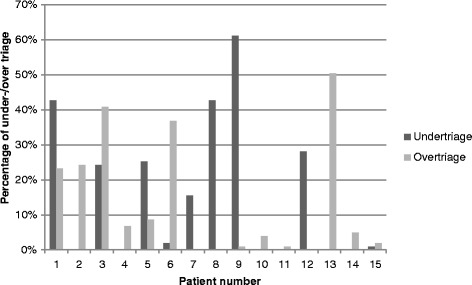
Over triage and under triage in the different patient scenarios immediately after the intervention

The median pretest score was 9 [8–10] and individual participant accuracy ranged from 4 to 12 of a maximum 15 points. The median post-test score was 10 [9–12] with individual accuracy ranging from 4 to 14 points, a significant improvement (*P* < 0.01). Over and under triage for the test at baseline was 23 and 18 %, respectively. Over and under triage for the test after the training was 14 and 17 %, respectively (Fig. 3).

### Direct instruction versus trauma cards

There were 40 participants in the trauma card session and 46 participants in the direct instruction session. Triage improvement score (post-test score—pretest score) for the trauma card group was 1.5 [0–3] compared with 2 [0–3.5] for the direct instruction group. This difference was not significant (*P* > 0.05).

### Follow-up

Fifty-one participants (59 %) completed the follow-up test. The median follow-up score was 10 [9–11] with individual triage accuracy ranging from 7 to 15. This difference was not significant compared with the post-test scores (*P* > 0.05). Because no significant difference between post test and follow-up, pretest was also tested against follow-up scores. There was significant increase in triage accuracy (*P* < 0.05). No difference regarding follow-up score between the trauma card and direct instruction groups was seen (*P* > 0.05). Over and under triage at follow-up was 17 and 15 %, respectively. Previous experience of multi-casualty incidents, years in service, level of education or age did not have any measurable effects on triage accuracy after the training or skill retention 6 months later.

## Discussion

In this study, we found that participants who were taught SALT triage during one hour made a significant improvement when comparing pretest scores with post-test scores. This indicates that 1 hour of triage training is sufficient to learn the basic skills of triage and this finding corroborates previous studies on SALT [15, 16] as well as studies using the Simple Triage And Rapid Treatment (START) algorithm [17, 18]. At the follow-up 6 months later, no significant deterioration in triage accuracy was found compared with the post-test assessment and was still higher than pretest. However, the loss of 35 participants (41 %) to the follow-up test does introduce uncertainties in the post-test and follow up analysis. The results are in contrast with an earlier study on the triage algorithm of the Smart Incident Command System from TSG Associates [19] and indicates that SALT triage accuracy persists 6 months after the intervention when trained in this way.

With regard to the use of trauma cards or direct instruction, the results showed no significant difference in triage accuracy compared to direct instruction. This makes the choice between trauma cards and direct instruction a matter for the instructor. However, it would be interesting to compare video instruction with no student interaction versus trauma cards. This may indicate whether earlier results [19] of deteriorating skills 6 months after an intervention depend on different triage algorithms or the actual training model. Even though no significant difference was seen in the present study, trauma cards can be a complement to full-scale exercises [20] and may involve and engage the participants to a high degree.

The absence of any significant difference in improvement with age, years as prehospital care provider, participation in MCIs and education indicates that SALT triage can be taught successfully irrespective of background. Further studies are needed to establish if a longer training intervention or the use of other triage systems might be beneficial for skill acquisition or retention outcome.

The high rates of under triage (16 %) compared with earlier studies [16, 21] could be attributable in part to the subjective “minor injuries only” decision point. Patient number nine whose injuries should not be regarded as minor represents 25 % of the under triage score (Fig. 3). There are currently no guidelines on what constitutes minor injuries, which makes it difficult to foresee how this could affect patient outcome. The relatively large decline in over triage but unchanged under triage might depend on participants being unused to classify patients to expectant as this category seldom is used in Sweden. Instead, classifying them as immediate resulting in high over triage.

The decisions of first responders are crucial and the goal in all casualty incidents is to give the right priority to the right patient to be transported to the right level of care all within the right time [9]. Correct triage in an MCI is one of the most important factors to minimize mortality and morbidity [4, 22]. Therefore, it is important that all first responders know the basics of on-scene triage and can identify the most severely injured. An earlier study has shown that education is a key factor to achieve this [23]. Firemen with prehospital experience combined with only 1 hour of triage training can perform accurate triage assessment and maintain those skills over time. However, further studies of assessment on scene and how this is related to patient outcome are needed. The present study indicates that 1 hour of triage training may be enough to learn and improve skills comparable with earlier studies [15–18] and that the skills may be retained for at least 6 months after the intervention. As with many simulations it is difficult to estimate the external validity of the study. It might have benefitted from testing triage performances using moulaged actors but as this had reduced the number of participants a written test was chosen.

Future research will help to determine how to extend the application of trauma cards. This study used one model combining ETS trauma cards with a short triage lecture; however, trauma cards can be used in many other combinations of exercise settings.

### Limitations

This study uses paper-based patient scenarios and the findings may not be the same as those for a real MCI. The study was also limited to firemen and the results may not be generalizable to other types of responders. The firemen were recruited through their superiors and the study took place on duty. Although everyone was informed verbally and in written form that participation in the study was voluntary, we do not know how the firemen were encouraged to participate. It remains unknown how triage skill accuracy in simulations translate to potential clinical effects in a real incident.

## Conclusions

One hour of triage training with the SALT Mass Casualty Triage Algorithm was enough to significantly improve triage accuracy in both groups of firemen with sustained skills 6 months later. Therefore, we propose that firemen should be well trained in triage as they are frequently first on scene at incidents. This may improve casualty outcome in real incidents as the time to definitive care is crucial to minimize mortality and morbidity. Despite the absence of a significant difference in triage accuracy improvement, trauma cards may still be useful to engage and involve participants in their own learning process. This aspect, as well as the efficacy and validity of trauma cards for disaster management training should be tested in future studies.

## References

[1] Schenk E, Wijetunge G, Mann NC, Lerner EB, Longthorne A, Dawson D (2014). Epidemiology of mass casualty incidents in the United States. Prehosp Emerg Care.

[2] American College of Surgeons. About advanced trauma life support. 2013. http://www.facs.org/trauma/atls/history.html. Accessed 22 Nov 2013.

[3] Frykberg ER (2005). Triage: principles and practice. Scand J Surg.

[4] Waeckerle JF (1991). Disaster planning and response. N Engl J Med.

[5] Debacker M, Hubloue I, Dhondt E, Rockenschaub G, Ruter A, Codreanu T (2012). Utstein-style template for uniform data reporting of acute medical response in disasters. PLoS Curr.

[6] Johnson DW, Johnson RT, Smith KA (1998). Cooperative learning returns to college. Change.

[7] Pourshanazari A, Roohbakhsh A, Khazaei M, Tajadini H (2013). Comparing the long-term retention of a physiology course for medical students with the traditional and problem-based learning. Adv Health Sci Educ.

[8] Lampi M, Vikstrom T, Jonson CO (2013). Triage performance of Swedish physicians using the ATLS algorithm in a simulated mass casualty incident: a prospective cross-sectional survey. Scand J Trauma Resusc Emerg Med.

[9] National Association of Emergency Medical Technicians (U.S.). Pre-hospital trauma life support committee., American College of Surgeons. Committee on Trauma (2011). PHTLS: Prehospital Trauma Life Support.

[10] Jenkins JL, McCarthy ML, Sauer LM, Green GB, Stuart S, Thomas TL (2008). Mass-casualty triage: time for an evidence-based approach. Prehosp Disaster Med.

[11] Lerner EB, Schwartz RB, Coule PL, Weinstein ES, Cone DC, Hunt RC (2008). Mass casualty triage: an evaluation of the data and development of a proposed national guideline. Disaster Med Public Health Prep.

[12] Kahn CA, Schultz CH, Miller KT, Anderson CL (2009). Does START triage work? An outcomes assessment after a disaster. Ann Emerg Med.

[13] Helsedirektoratet (2013). Nasjonal veileder for masseskadetriage [in norwegian].

[14] Lennquist S (2009). Katastrofmedicin.

[15] Deluhery MR, Lerner EB, Pirrallo RG, Schwartz RB (2011). Paramedic accuracy using SALT triage after a brief initial training. Prehosp Emerg Care.

[16] Lerner EB, Schwartz RB, Coule PL, Pirrallo RG (2010). Use of SALT triage in a simulated mass-casualty incident. Prehosp Emerg Care.

[17] Sanddal TL, Loyacono T, Sanddal ND (2004). Effect of JumpSTART training on immediate and short-term pediatric triage performance. Pediatr Emerg Care.

[18] Risavi BL, Salen PN, Heller MB, Arcona S (2001). A two-hour intervention using START improves prehospital triage of mass casualty incidents. Prehosp Emerg Care.

[19] Risavi BL, Terrell MA, Lee W, Holsten DL (2013). Prehospital mass-casualty triage training-written versus moulage scenarios: how much do EMS providers retain?. Prehosp Disaster Med.

[20] Wakasugi M, Nilsson H, Ruter A (2013). Figurant cards as a tool to link live field and virtual simulation exercises in disaster medical training. J Clin Simul Res.

[21] Cone DC, Serra J, Kurland L (2011). Comparison of the SALT and smart triage systems using a virtual reality simulator with paramedic students. Eur J Emerg Med.

[22] Debacker M, Ullrich C, Van Utterbeeck F, Dejardin E, Dhondt E, Hubloue I. Triage decreases the mortality in a simulated road traffic MCI scenario. Amsterdam: 8th European Congress on Emergency Medicin. 2014. Accessed date 28/9-1/10 http://archive2014.eusemcongress.org/upload/finalok.pdf.

[23] Considine J, Botti M, Thomas S (2007). Do knowledge and experience have specific roles in triage decision-making?. Acad Emerg Med.

